# Should Early Use of Chemotherapy Be Avoided in the Treatment of Metastatic Prostate Cancer?

**DOI:** 10.31662/jmaj.2021-0207

**Published:** 2021-12-24

**Authors:** Takahiro Kimura

**Affiliations:** 1 Department of Urology, The Jikei University School of Medicine, Tokyo, Japan

**Keywords:** prostate cancer, castration-resistant, docetaxel

The therapeutic strategy for advanced prostate cancer has dramatically changed recently. Previously, docetaxel was the only drug with evidences showing improved overall survival (OS) in metastatic castration-resistant prostate cancer (CRPC). However, androgen receptor pathway inhibitors (ARPI), such as abiraterone acetate and enzalutamide, cabazitaxel, radium-223, and olaparib, have emerged since the 2010s. In addition, early use of docetaxel or ARPIs with androgen deprivation therapy for castration-sensitive prostate cancer has also shown significant survival benefit over the last several years. Under certain circumstances, appropriate sequence of the drugs has been one of the important clinical questions in this field.

Matsumoto et al retrospectively compared the efficacy of second-line ARPI, docetaxel, and radium-223 in patients with CRPC who received ARPI as first-line treatment. The results indicated that docetaxel showed significantly better progression-free survival (PFS) than second-line ARPI and radium-223, and OS was not different among them ^(1)^. The results of this study were consistent with those of previous retrospective studies ^(2), (3)^. The cause of the difference in efficacy between drugs as second-line treatment is thought to be cross-resistance among ARPIs, which was reported in several prospective and retrospective studies in metastatic CRPC. In the prospective randomized crossover trial to assess the optimal sequencing of enzalutamide and abiraterone acetate, time to prostate-specific antigen (PSA) progression of second-line enzalutamide and abiraterone acetate was 3.5 and 1.7 months ^(4)^, respectively, which was consistent with the results of Matsumoto’s study; median PFS of second-line ARPI was 2.8 months.

Another interesting finding of Matsumoto’s study was that PFS of second-line docetaxel was significantly better than that of second-line ARPI even in the subgroup with maximum PSA response of first-line ARPI > 50%. Generally, docetaxel is recommended as second-line treatment after progression of first-line ARPI. However, second-line ARPI is thought to be effective in patients who show a good response to first-line ARPI. Nevertheless, the results of this study revealed that the efficacy of second-line ARPI was limited even in good responders of first-line ARPI. Therefore, using drugs with a different mechanism of action in appropriate timing is an important treatment strategy for metastatic CRPC.

According to the evidences so far, including this study, docetaxel should be recommended as second-line treatment for metastatic CRPC patients who received first-line ARPI. However, docetaxel is selected as second-line treatment only for limited patients in real-world clinical practice. In a retrospective study of the Veterans Health Administration database, including 3,174 metastatic CRPC patients treated with first-line ARPI from 2014 to 2018, 1229 and 1945 patients were treated with enzalutamide and abiraterone acetate, respectively. Among then, 45% and 54% of patients were received second-line therapy during the observation period, but only 6% and 9% were administrated docetaxel, whereas 23% and 26% were administrated second-line ARPI, respectively ^(5)^. Prostate cancer is relatively slow growing even when in the metastatic state. In addition, both ARPI and chemotherapy are similarly effective. On the other hand, chemotherapy is more toxic than ARPI due to severe adverse events, such as neutropenia, febrile neutropenia, alopecia, and neuropathy. Thus, patients tend to prefer ARPIs than chemotherapy. However, the efficacy of second-line ARPI is limited and most of the patients need to change the treatment a couple of months later. In addition, patients might lose the chance to receive chemotherapy due to progression of the disease in later-line treatment sequences. Clinicians must explain the fact and prospect of the treatment strategy to patients during the early phase of treatment.

Several clinical trials of new drugs for metastatic CRPC with different modes of action are ongoing. Under such circumstances, we must provide an appropriate therapeutic strategy based on the evidences. In addition, further studies are required to assess whether the current evidences of CRPC can be adapted to CRPC after combined treatment of ARPI and androgen deprivation therapy for metastatic castration-sensitive prostate cancer.

## Article Information

### Conflicts of Interest

Takahiro Kimura is a paid consultant/advisor of Astellas, Bayer, Janssen, and Sanofi.

### Author Contributions

TK wrote this manuscript.

### Approval by Institutional Review Board (IRB)

NA
